# Minimal change nephrotic syndrome after stem cell transplantation: a case report and literature review

**DOI:** 10.1186/1752-1947-1-121

**Published:** 2007-10-30

**Authors:** Sandra Silva, José Maximino, Rui Henrique, Ana Paiva, Jorge Baldaia, Fernando Campilho, Pedro Pimentel, Alfredo Loureiro

**Affiliations:** 1 Department of Nephrology, Portuguese Oncology Institute, Porto, Portugal; 2 Department of Pathology, Portuguese Oncology Institute, Porto, Portugal; 3 Department of Bone Marrow Transplant, Portuguese Oncology Institute, Porto, Portugal; 4 Department of Pathology and Molecular Immunology, Instituto Ciências Abel Salazar, Oporto University, Porto, Portugal

## Abstract

Graft-versus-host disease is one of the most frequent complications occurring after haematopoietic stem cell transplantation. Recently, renal involvement has been described as a manifestation of chronic graft-versus-host disease. Immunosuppression seems to play a major role: clinical disease is triggered by its tapering and resolution is achieved with the resumption of the immunosuppressive therapy. Prognosis is apparently favourable, but long term follow up data are lacking.

We report a case of a 53-year-old man who developed nephrotic syndrome 142 days after allogeneic stem cell transplantation for acute myeloid leukaemia. Onset of nephrotic syndrome occurred after reduction of immunosuppressants and was accompanied by manifestations of chronic graft-versus-host disease. Histological examination of the kidney was consistent with Minimal Change Disease. After treatment with prednisolone and mycophenolate mofetil he had complete remission of proteinuria and improvement of graft-versus-host disease. Eighteen months after transplantation the patient keeps haematological remission and normal renal function, without proteinuria.

Since patients with chronic graft-versus-host disease might be considered at risk for development of nephrotic syndrome, careful monitoring of renal parameters, namely proteinuria, is advisable.

## Case presentation

A 53-year-old hypertensive man with adenomatous colonic polyposis was found to have acute myeloid leukaemia (AML) M_2 _after diagnostic workup of rectal bleeding.

He had consanguine parents. His father died at 90 and his mother at 76 years. They had no known colonic polyps or history of colonic cancer. He had 3 brothers: two healthy, with 60 and 61 years and a 56 year-old with AML M_3 _who has been submitted to a total colectomy due to adenomatous colonic polyposis. Colonoscopic screening was, unsuccessfully, attempted in the patient's offspring. At the time of AML diagnosis, March 2005, no chromosomal abnormalities were detected in the leukaemic blasts. First complete remission was achieved in April 2005 with Cytarabin/Idarubicin. A relapse was identified 4 months later and the patient was treated with Carboplatin/Etoposide. After this second remission, he underwent peripheral blood stem cell transplant (PBSCT) from an HLA-identical brother in December 2005. Reduced-intensity conditioning was performed with Fludarabine/Busulfan and graft-versus-host disease (GVHD) prophylaxis with Cyclosporine (CsA)/Methotrexate. Platelet and neutrophil recovery to > 20 × 10^9^/L and > 0.5 × 10^9^/L respectively, were achieved on days +10 and +18, correspondingly. A 100% donor chimerism was confirmed. Regimen haematological toxicities were minimal, but he developed acute GVHD (skin +3/global II, of the 1994 Consensus Conference on Acute GVHD Grading) with maculopapular rash on day +20 after transplant. Complete response to prednisolone was reached. A CMV infection was identified on day +30 and treated with valganciclovir.

On day +142 the patient was admitted with anasarca. He claimed of dry, painful eyes and xerostomia that started a few weeks ago. He was afebrile, with blood pressure of 140/92 mmHg, pulse of 80 bpm, with no respiratory distress. He had no organomegalies but facial and lower limbs oedema was identified. Pigmented lesions (poikiloderma), lichen planus-like eruptions, sclerotic features characterized by thickened skin and maculopapular rash in the palmar and facial regions were present.

At this time point his medication was: CsA 50 mg q12h, prednisolone 30 mg qod, enalapril 20 mg qd, bisoprolol 5 mg qd, sulfamethoxazole/trimethoprim 960 mg three times a week, acyclovir, mianserine, folate and magnesium. CsA and prednisolone have been progressively tapered, as planned.

He had *de novo *severe proteinuria (14 g/24 h), hypoalbuminemia (2 g/dL), hypercholesterolemia (243 mg/dL), acute renal failure (creatinine 2 mg/dL; urea 102 mg/dL) and an erythrocyte count of 8 cells/hpf, leucocyturia and hyaline-granular casts in the urine test. A diagnosis of nephrotic syndrome (NS) was rendered.

Hepatic enzymes were increased: AST 173 U/L, ALT 253 U/L, alkaline phosphatase 505 U/L, GGT 1637 U/L, 5'nucleotidase 244 U/L and total bilirubin 2.7 mg/dL. Hematologic findings included anemia (7 g/dL), thrombocytopenia (43 000) and reticulocytosis (7%); the leukocyte count was normal. A peripheral blood smear showed no evidence of microangiopathy. Increased serum lactic dehydrogenase, indirect bilirubin and low haptoglobin were also found. Direct Coomb's test and anti-platelet antibodies were positive. Prothrombin time, activated partial thromboplastin time and fibrinogen were within reference range. Antinuclear antibody, anti-ds DNA, cryoglobulin and hypocomplementemia were not present. Serum immunoglobulins were within normal limit. Tests for hepatitis B virus surface antigen, hepatitis C and HIV antibody, CMV IgM, CMV pp65 antigen, EBV IgM and serological tests for adenoviruses and parvovirus B19 were negative. Culture for bacteria and fungi revealed no microbiological growth. Serum/urinary immunoelectrophoresis disclosed no monoclonal proteins. CsA trough level was 97 ng/mL. Abdominal ultrasonography and chest X-rays were normal. A pelvic magnetic resonance was normal and excluded renal venous thrombosis. Relapse of AML was excluded.

**Figure 1:**
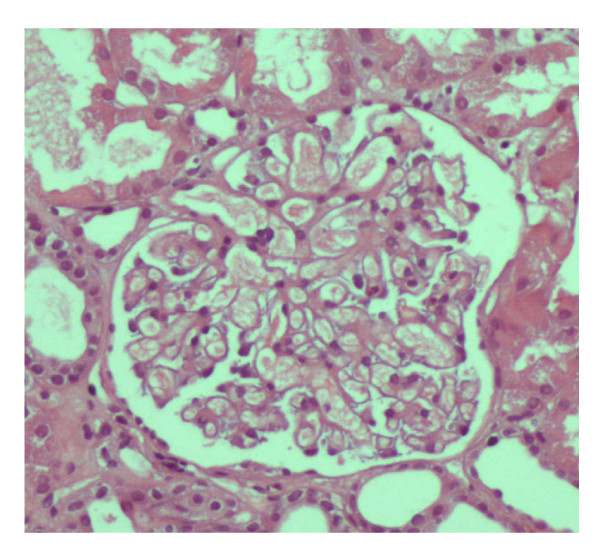
**Renal biopsy specimen **(HE): An essentially normal glomerulus, with open capillary lumens and only minimal hypercellularity.

**Figure 2:**
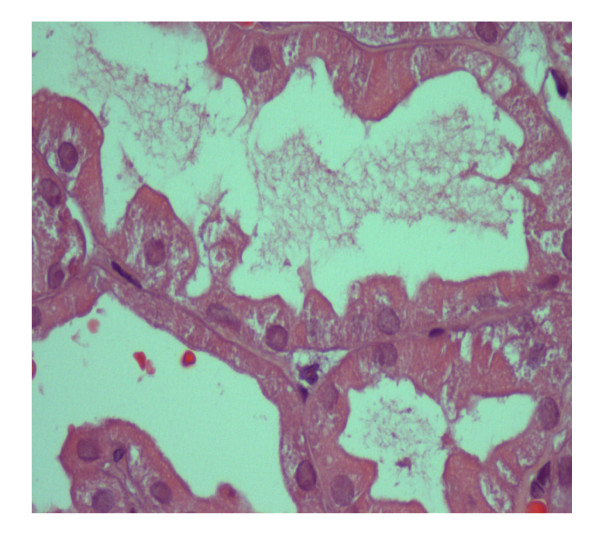
**Renal biopsy specimen **(HE): focal loss of tubular epithelial cells and presence of cellular debris inside of the tubules, consistent with acute tubular necrosis; no interstitial fibrosis was present. Immunofluorescence (not shown) was negative.

**Figure 3:**
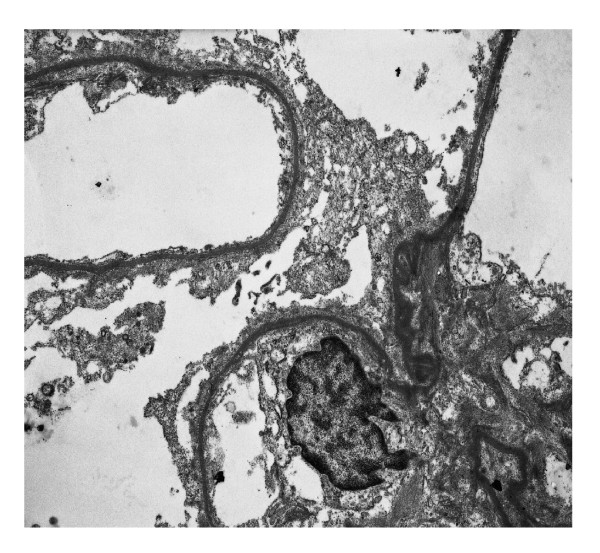
**Renal biopsy specimen **(Electron micrograph × 10000): Epithelial cells effaced along the glomerular basement membrane.

CsA was withdrawn and a percutaneous renal biopsy was performed. Pathological examination disclosed 15 glomeruli, with patent lumina and absence of increased mesangial cellularity or matrix deposition (figure 1). Morphologic features of acute tubular necrosis (figure 2) were found without evident hyaline thrombi. No immune deposits were detected by fluorescence microscopy. Ultrastructural analysis disclosed only diffuse podocyte fusion (figure 3). Thus, a diagnosis of Minimal Change Disease and acute tubular necrosis was revealed.

**Figure 4:**
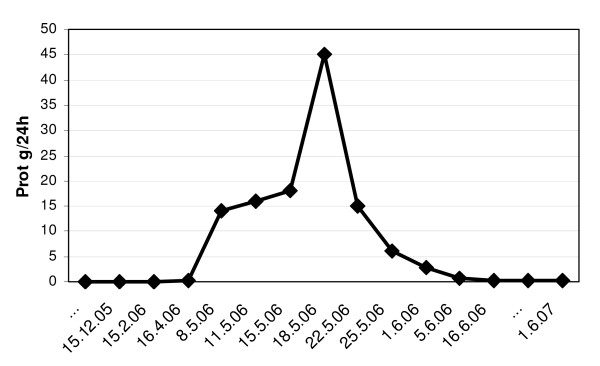
Degree of proteinuria before and after PBSCT.

Treatment with high dose corticosteroids (i.v. methylprednisolone 1 g × 3 days followed by oral prednisolone 1 mg/kg/day) and mycophenolate mofetil (MMF 1 g bid) was started and complete remission of proteinuria was achieved 4 weeks later (figure 4). NS was complicated by thrombosis in the femoropopliteal veins of the right leg (three weeks after hospital admission) and the patient was placed on anticoagulant therapy. During hospitalization he developed new onset of diplopia. A right petroclival meningioma was diagnosed by magnetic resonance. The management was conservative, with clinical surveillance. At the present time he has no diplopia. A gastroenterology consultation concluded that the patient had attenuated familial adenomatous polyposis. Colonic polyps showed regression after therapy with sulindac. Search for mutations at the *APC *and *MYH *loci was negative.

Currently, 18 months after transplantation, the patient maintains haematological remission, normal kidney and hepatic function and urinary protein loss of less than 0.3 g/24 h. He is still on immunosuppressive therapy with MMF 1 g bid and prednisolone 10 mg every other day due to ocular GVHD.

## Background

Haematopoietic stem cell transplantation (HSCT) is an increasing therapeutic option for a variety of malignant and non malignant haematological diseases. Renal complications associated with this procedure have an ominous prognosis [1]. Imai et al [1] classified HSCT-related nephropathy into three groups according to the time of onset. During the early post transplant period sepsis, tumour lysis syndrome, hepatic venooclusive disease and nephrotoxicity may cause acute renal failure. Middle-onset includes haemolytic uremic syndrome/thrombotic thrombocytopenic purpura. After 6 months multifactorial chronic renal failure is a recognized sequela of HSCT.

Recently, nephrotic syndrome (NS) after HSCT has been reported, usually associated with graft-versus-host disease (GVHD) [2]. Imai group [1] described 8 NS cases in 2136 stem cell recipients. An extensive literature review identified 46 case reports [2]; recently, 4 further cases [3] were described.

GVHD is the most common late complication of HSCT, occurring in 60–80% of long term survivors and causing significant morbidity and mortality [4]. In the past, any manifestation of GVHD that was present at 100 or more days after HSCT was arbitrarily defined as chronic GVHD. This definition had been questioned and the current consensus of the National Institutes of Health [5] is that clinical manifestations, and not time, determine whether the clinical syndrome is considered acute or chronic. GVHD resembles autoimmune disorders such as Sjögren syndrome but the physiopathology is far from clear. The skin, the liver, the gut and the eyes are frequently damaged; conversely, kidney [2] and haematopoietic system [6] are seldom involved. The main risk factor for chronic GVHD is HLA mismatch. Its incidence is also higher in older recipients, gender mismatch (female donor to male recipient), allogeneic versus autologous and PBSCT versus bone marrow transplantation (BMT) [7].

Pathological diagnosis of GVHD-related NS is essentially Membranous Nephropathy (MN) in two thirds and Minimal Change Disease (MCD) in nearly one quarter of patients [2,8]. However, as recently confirmed, a wide spectrum of renal diseases can be found: focal segmental glomerulonephritis [2] including the "tip variant" [9], diffuse proliferative glomerulonephritis [10], IgA nephropathy [11], membranoproliferative glomerulonephrities [3] and antineutrophil cytoplasmic antibody-associated glomerulonephritis [12].

Typically, a temporal relation between NS, cessation/tapering of immunosupressants and simultaneous GVHD is seen. NS generally, responds well to immunosupressive therapy, but this seemingly good prognosis has to be weighed against our limited knowledge in this setting.

## Discussion

Herein, we present a case of MCD at +142 day after PBSCT. MCD is a rare complication of HSCT, with only 10 cases reported thus far. In these 10 patients, the underlying predominant haematological disease was AML; when overall HSCT related NS are considered the most frequent diagnosis was Chronic Myeloid Leukaemia.

Almost all reviewed cases were myeloablative transplants. However, a cohort study [13] identified nonmyeloablative HSCT as a risk factor for NS. In this six-year experience, seven of 163 patients (4.3%) after nonmyeloablative transplant, as opposed to none of 118 patients after myeloablative transplant, developed this complication.

Peripheral blood compared to bone marrow cells, is increasingly used as a source of haematopoietic stem cells. Some studies suggested an increased GVHD incidence with the former, which may be explained by the transfer of a greater T cell dose with the PBSCT. In a report from Colombo et al [14] patients grafted with peripheral stem cells have a 24% and those with bone marrow have a 3% probability of developing NS. Consequently, in the near future, we might expect an increased number of glomerular diseases after HSCT.

Seconi et al [15] recently proposed an association between NS following GVHD and an increased production of Tumor Necrosis Factor alpha (TNFα) by donor T cells. They established that excessive TNFα and Interferon gamma (IFNγ) were implicated in the development of skin and gut GVHD and that high TNFα production had a role in the development of idiopathic NS. Consequently, TNFα might be seen as a target for future therapeutic options.

MCD usually appeared early after HSCT (median 8 versus 14 months for MN) [2]. The most common clinical manifestations were fatigue and oedema [14]. The median presenting plasma creatinine and proteinuria values were 1.2 mg/dL and 13 g/day, respectively [14]. Auto antibodies and serum complement levels were typically normal.

The most striking feature of HSCT related NS was the association with GVHD. Indeed, the majority of patients reported had evidence of acute and/or chronic GVHD. GVHD was simultaneous in 3 and preceded NS in 4 of the 10 MCD patients [2]. Nevertheless, there are at least 3 cases of NS after HSCT without evidence of GVHD.

Changes in immunosuppressant doses were reported in the majority of patients. The time course between discontinuation and/or tapering of immunosuppressants and NS onset suggests the former as a risk factor.

Therapeutic options included mostly steroids and cyclosporine, but other agents like cyclophosphamide, tacrolimus, MMF and rituximab had been used [12]. Nonetheless, so far, ideal treatment, dose, duration or target levels of immunosuppressive medications is still unknown.

Similar to idiopathic MCD, almost all patients achieved complete remission and response was generally reached after 8–10 weeks of treatment [1]. However, concerning MN, prognosis does not seem so favourable: only 27% showed complete and 62% partial remission, in Ratanatharathorn's study [4]. Furthermore, a poor response and a high propensity for progressive renal failure were described by others [10]. In addition, the majority of the authors do not make substantial comments on long term follow-up or on the potential deleterious effect of increased immunosupression. Conversely, Kemper and colleagues [3], described 3 MN cases with a sustained remission of the NS.

Among the reported post-HSCT nephrotic cases, haemolytic anaemia and immune thrombocytopenia are uncommon. However, autoimmune cytopenias are part of the chronic GVHD spectrum and are caused by antibodies [5,6] of donor origin directed against the recipient red cell/platelet antigens. Among patients who developed chronic GVHD, Godder and co-authors [6] reported five cases (28%) presenting with haemolytic anaemia (HA) with or without thrombocytopenia. Along with a mean haemoglobin level of 7.1 g/dL, patients had an increased reticulocyte count, a mild raised lactic acid dehydrogenase, an increased indirect bilirubin and a positive Coombs' test. Our patient showed similar findings. The evidence of an immune-mediated process (as suggested by the positive Coombs' test and platelet antibody) and the response to intensive immunosuppressive therapy with simultaneous regression of chronic GVHD lesions supported our diagnosis of involvement of the haematopoietic system by GVHD.

Acute renal failure in our patient was due to acute tubular necrosis. A reduction in glomerular filtration rate may be seen in MCD. Aetiologies include ischemic injury, interstitial oedema with tubular collapse, excessive diuretic use or anti-inflammatory drugs. Renal vein thrombosis is another possible cause, but was excluded in our case. Biopsy specimens confirmed changes similar to that seen in postischemic acute tubular necrosis.

Although a liver biopsy and a Schirmer test were not performed we assumed hepatic and ocular involvement by chronic GVHD due to the fact that concomitant and distinctive manifestations in the skin were present. Fading of hepatic and ocular lesions concomitant with vanishing of the GVHD with immunosuppression do support our hypothesis.

Here, we reported a case of an allogeneic PBSCT recipient who developed chronic GVHD according to Filipovich's classification [5]. Cutaneous (poikiloderma and sclerotic features), ocular (xerophthalmia), hepatic (cholestasis), hematological (autoimmune cytopenias) and renal (nephrotic syndrome) involvement by GVHD was identified. To the best of our knowledge this is the first reported case of GVHD with involvement of both the kidney and the haematopoietic system, two exceptional manifestations of GVHD.

## Conclusion

NS is a well-characterized, although unusual complication of HSCT that occurs in association with GVHD flares after taper of immunosuppression. The reviewed literature suggests that glomerular lesions actually represent renal GVHD, but pathophysiology is unclear and warrants further investigation. Longer follow-up of larger cohorts is mandatory to identify definitive HSCT related NS risk factors.

Data on glomerulopathies, in addition to renal function, should be routinely collected and recorded as part of BMT complications.

## Competing interests

The author(s) declare that they have no competing interests.

## Authors' contributions

SS collected the patient's data, performed the literature review on similar cases, wrote the manuscript. JM contributed to writing the manuscript. RH performed the histological examination of the kidney, was a major contributor in writing the manuscript. AP was a major contributor in writing the manuscript. JB contributed to writing the manuscript. FC analyzed and interpreted the patient data regarding the haematological disease and the transplant. PP analyzed the patient data regarding the haematological disease and the transplant. AL gave final approval of the version to be submitted for publication

## Consent

Written informed consent was obtained from the patient for publication of the case-report.
